# Layer combination of similar infill patterns on the tensile and compression behavior of 3D printed PLA

**DOI:** 10.1038/s41598-025-94446-8

**Published:** 2025-04-06

**Authors:** Menna G. Aboelella, Samy J. Ebeid, Moustafa M. Sayed

**Affiliations:** Design and Production Engineering Department, Faculty of Engineering, Ain Shams University, Cairo, Egypt

**Keywords:** Polylactic acid, Combined infill patterns, Polymer, 3D-printing, Infill structure, Additive manufacturing, Fused deposition modeling (FDM), Mechanical engineering, Materials science, Soft materials, Techniques and instrumentation

## Abstract

With the growing popularity of 3D-printed products, material consumption has been a major concern in additive manufacturing in recent years. Choosing the infill structure and the printing parameters for an application can be challenging for product designers and engineers, which can lead to reduced material and increased cost savings while maintaining product functioning.

This study investigates the mechanical behavior of 3D-printed PLA structures by exploring the influence of multi-layer infill patterns on tensile and compressive strength. Three common infill patterns (triangular, grid, and honeycomb) were evaluated at 20% and 50% densities. A novel approach was employed, incorporating specimens with single-, two-, and four-layer same pattern combinations, where subsequent layers were rotated 180 degrees to enhance interlayer bonding. Results demonstrated significant improvements in both tensile (up to 64%) and compressive strength (up to 47%) for two-layer structures compared to single-layer counterparts. The findings provide valuable insights into optimizing infill design and layer configurations for improved tensile and compressive strength and material efficiency in 3D-printed structures. This research highlights the potential for optimizing 3D-printed part performance through strategic multi-layer infill design, offering a pathway toward reduced material consumption and enhanced mechanical properties in additive manufacturing.

## Introduction

In recent years, one of the main challenges among academic and industrial centers has been accelerating manufacturing processes^1^. Rapid prototyping (RP) technologies have emerged in this regard. RP is primarily based on additive manufacturing (AM), which has gained popularity across multiple disciplines^2^. Several types of AM have been developed and introduced thus far, such as fused deposition modeling (FDM), selective laser melting (SLM), and selective laser sintering (SLS). FDM is the most widely used technique among them, as noted in studies^3,4^.

For engineers, it is important to consider the limits of each AM method. The FDM method is widely recognized as the most employed approach among them. This is because it may employ a wide variety of raw materials and is far easier and more affordable to use than other techniques^5^. The main benefit of FDM is that it can produce hollowed-out sections, which can result in significant weight and material savings. However, it is important to remember that the layered structure and printing method can have a big impact on the characteristics of the part^6^.

During printing, the temperature of the printing chamber affects how strongly adjacent fibers bond to one another in the part. Furthermore, the final properties of the part are influenced by the bonding strength between the rods^7^. Most studies conducted on the FDM process have concentrated on increasing mechanical stiffness or shortening the manufacturing time^8^. Many studies have been carried out to shorten the time required for FDM manufacturing. To maximize feed speeds during printing or decrease nozzle displacement, some researchers have suggested deposition strategies^9^. Parts of intricate external forms as well as intricate internal geometries (infill), like cellular or lattice structures, can be produced using the FDM process.

The primary advantage of employing a lattice structure is that it uses less material and takes less time to complete while maintaining the strength of the part, which lowers costs^10^. Slicing software is typically used to create the infill design, which is consistent throughout the structure. The percentage of infill has an impact on the weight of the part, mechanical stiffness, and printing time. Thus, optimizing the strength-to-weight ratio is one of the primary goals of the study^11^. Thermoplastics and polymer-based composites are the primary materials used in FDM, though a variety of materials are used, including low-temperature metal alloys and composites^12^. Build orientation, infill pattern, infill density, nozzle temperature, nozzle diameter, printing speed, and layer thickness are all important factors in producing a good mechanical product^13^. Unfavorable circumstances and low temperatures cause problems with printing processes like shrinkage and warpage^14^. It should be mentioned that determining Young’s modulus, tensile strength, and elongation at break is essential for isotropic and anisotropic engineering plastics to improve mechanical properties^15^. The disadvantage of the FDM process is that it tightens the gaps for better binding and creates support structures to prevent material drops. However, the surface texture of areas with supports attached to them is influenced by the support structure, and these areas become rough and of poor quality^16^.

The staircase effect, which results from layer binding behavior, is another flaw in parts that are printed using FDM^17^. The most important parameters that affect FDM product mechanical strength and quality are the infill density and infill pattern^18^. Shivani et al.^19^ observed that the infill density has a significant effect on the tensile strength values of 3D-printed PLA specimens. Tanveer et al.^20^ measured the PLA specimen’s impact strength at 50%, 70%, and 100% infill density and considered the mechanical properties to be directly proportionate to infill density. Chicos et al.^21^ showed that the infill density percentage has a major influence on mechanical properties. As the infill density increases, so does the tensile and flexural strength. Liu et al.^22^ showed that for Naylon-based material, the more mass the specimen has, the higher the compressive strength is. Li et al.^23^ extensively investigated the mechanical properties of TPMS structures, highlighting their potential for applications requiring high strength-to-weight ratios. However, research on the impact resistance of hybrid TPMS structures with varying connection geometries, particularly under high strain rate loading, remains relatively limited. Chen et al.^24^ investigating the influence of processing parameters on mechanical properties to achieve maximum compressive and flexural strength in the printed LRS structures.

Previous studies have investigated the mechanical performance of various infill patterns in 3D printing. Biroz et al. [25] showed that honeycomb and gyroid patterns can exhibit superior mechanical resistance compared to grid patterns. Aloyaydi et al.^26^ explored the impact of infill patterns on impact resistance and compressive strength, with triangular patterns demonstrating higher impact resistance and grid patterns exhibiting greater compressive strength. Podroužek et al.^27^ introduced and characterized novel 3D infill patterns inspired by nature and topology optimization, such as Gyroid, Schwarz D, and Schwarz P, demonstrating promising mechanical properties. Vos et al.^28^ investigated the influence of infill patterns on the print quality of dispensed materials, finding that different patterns significantly affect print speed, coverage, and surface roughness. valean et al.^29^ comprehensively characterizes the compressive behavior of 3D-printed PLA parts at large strains, revealing significant variations in properties across different infill patterns. Honeycomb demonstrated the best energy absorption. Rajiev et al.^30^ optimizes 3D printing parameters for PLA to improve mechanical properties and surface quality, utilizing the Taguchi method and SEM analysis.

While prior research has extensively investigated the effects of individual infill patterns and densities on the mechanical behavior of 3D-printed structures, the influence of multi-layer infill configurations, particularly those incorporating rotational offsets between successive layers, remains largely uncharted. Existing literature primarily concentrates on optimizing single-layer infill patterns to achieve desired mechanical properties. Most studies have focused on altering one or two process parameters to characterize their impact on mechanical performance. This study addresses this critical gap in the literature by investigating the effect of combining multiple layers of the same infill pattern with 180-degree rotations on the tensile and compressive strength of 3D-printed PLA specimens. This novel approach aims to enhance interlayer bonding and potentially improve overall part strength by mitigating the inherent weaknesses associated with single-layer structures, such as anisotropic behavior and reduced interlayer adhesion.

## Materials and methods

This study utilized polylactic acid (PLA), a widely employed biodegradable thermoplastic, for all 3D-printed specimens. An XYZ-“ULTIMAKER S5” 3D-printer equipped with a 0.4 mm nozzle was employed for the fabrication process. 3D-model slicing and parameterization were performed using UltiMaker Cura software. A 1.75 mm diameter white PLA filament^31^ was selected, balancing print quality, speed, and material compatibility. This filament choice ensures sufficient material flow and enables the creation of intricate details.

The printing process was carefully controlled with a layer height of 0.27 mm and a print temperature of 210 °C. Two infill densities were investigated: 20% and 50%. The 20% infill density prioritizes material efficiency and minimizes part weight, potentially leading to reduced stiffness and strength while improving printability and reducing printing time due to increased space between infill lines. The 50% infill density aims to achieve a balance between material efficiency and structural integrity, providing a reasonable level of mechanical performance while maintaining a relatively low material usage.

Triangular, grid, and honeycomb infill patterns were selected for their common usage, geometric simplicity, and ability to represent a diverse range of structural properties. This selection allows for a comprehensive investigation of the influence of infill patterns on the mechanical behavior of 3D-printed structures. After printing, samples were allowed to cool naturally on the build platform before removal.

### Design and manufacturing of samples

Dezaki et al.^32^ have previously discussed evaluating products with different patterns in each layer. When changing patterns, the primary considerations are building time, energy consumption, material utilization, strength, and surface quality.

In the present work, different infill patterns, triangular, honeycomb, and grid, have been studied to understand their impact on the 3D-printed structure. The tensile and compressive strength of 3D-printed structures are investigated according to ASTM D638 and ASTM D695 standards respectively. Infill densities of 20% and 50% were chosen for the specimens. The 3D printing software cannot accommodate samples with mixed patterns and layers directly only one pattern and one layer can be set on the software. So, to achieve the aim of this study, the three patterns and combined layers under study were inserted into machine software as different drawing files drawn by SolidWorks^®^ software, each for its sample category. All printed samples have the same thickness considered in each test standard to keep a fair comparison between the three categories of the samples for each mechanical test.

**Fig. 1:**
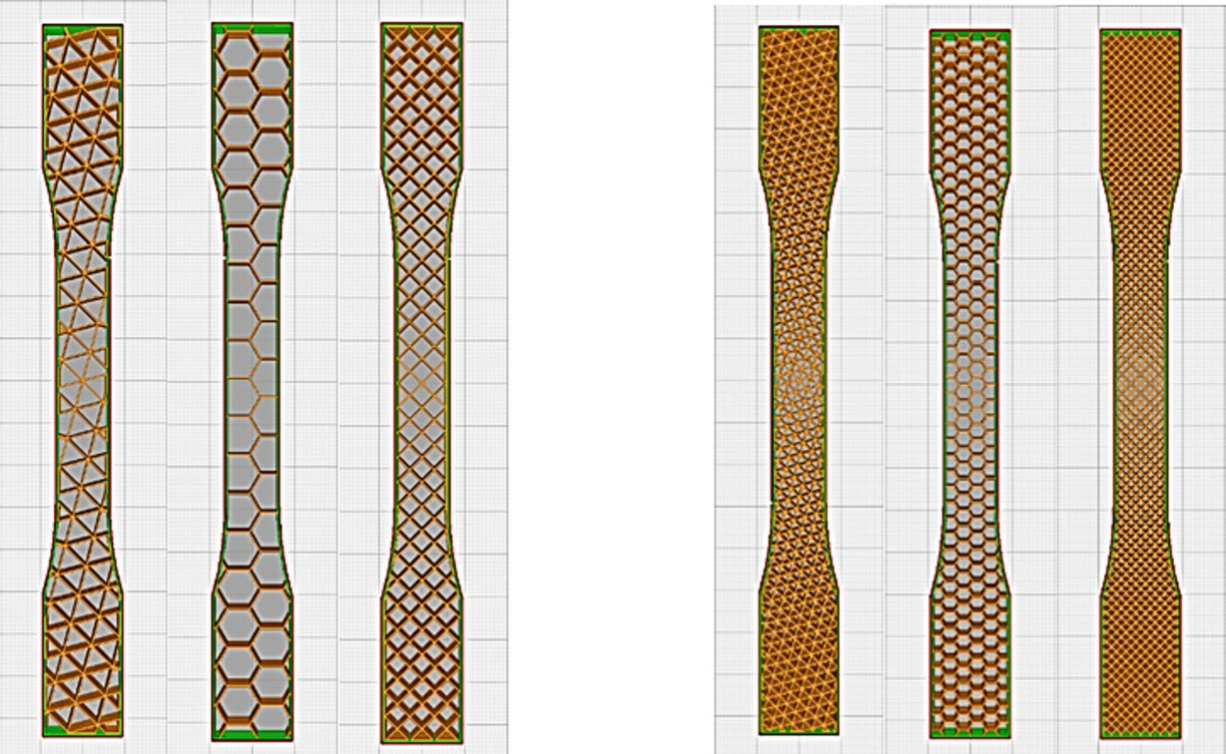
1st category samples at 20%&50% infill density (single layer).

The printed samples are classified into three categories. The first category is for the samples built with a single layer of one infill pattern. The samples were printed without top and bottom layers as shown in Fig. 1 for the 20% and the 50% infill density to keep the similar building configuration of the second and third categories.

(Ultimaker Cura software version 5.6.0)

**Fig. 2:**
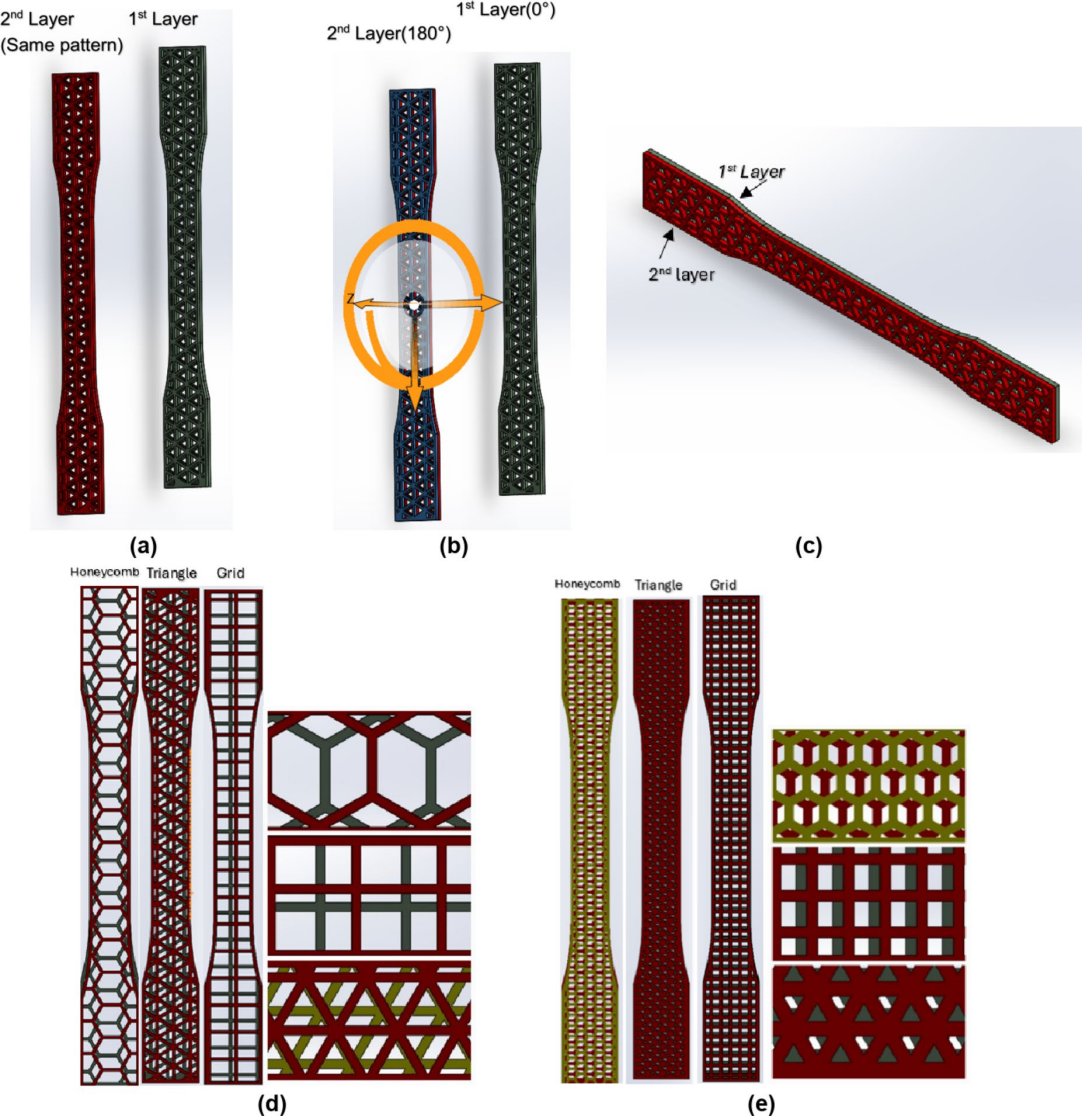
Combined layer samples in a similar pattern (two layers)(SolidWorks 2023) **(a)** Two layers sample: front and back views **(b)** Two layers sample: rotation of the second layer 180° relative to the first layer **(c)** Configuration of two layers sample (Final sample) **(d)** Combined samples with 20% infill density **(e)** Combined samples with 50% infill density.

The second category comprises specimens fabricated with two layers of the same infill pattern. Each specimen consists of two layers: a base layer with the chosen infill pattern (grid, triangular, or honeycomb) printed at a 0° orientation, followed by a second layer of the same infill pattern printed at a 180° rotation directly above the base layer. The 180-degree rotation of the second layer was chosen to maximize interlayer interlocking and enhance the mechanical properties of the specimens. This approach was selected for its simplicity, reproducibility, and potential to minimize anisotropic behavior. Figure 2 depicts the fabricated specimens for each of the three infill patterns (triangular, grid, and honeycomb) at 20% and 50% infill densities. A consistent linking pattern was employed between adjacent layers, resulting in a structurally uniform specimen along its length.

**Fig. 3:**
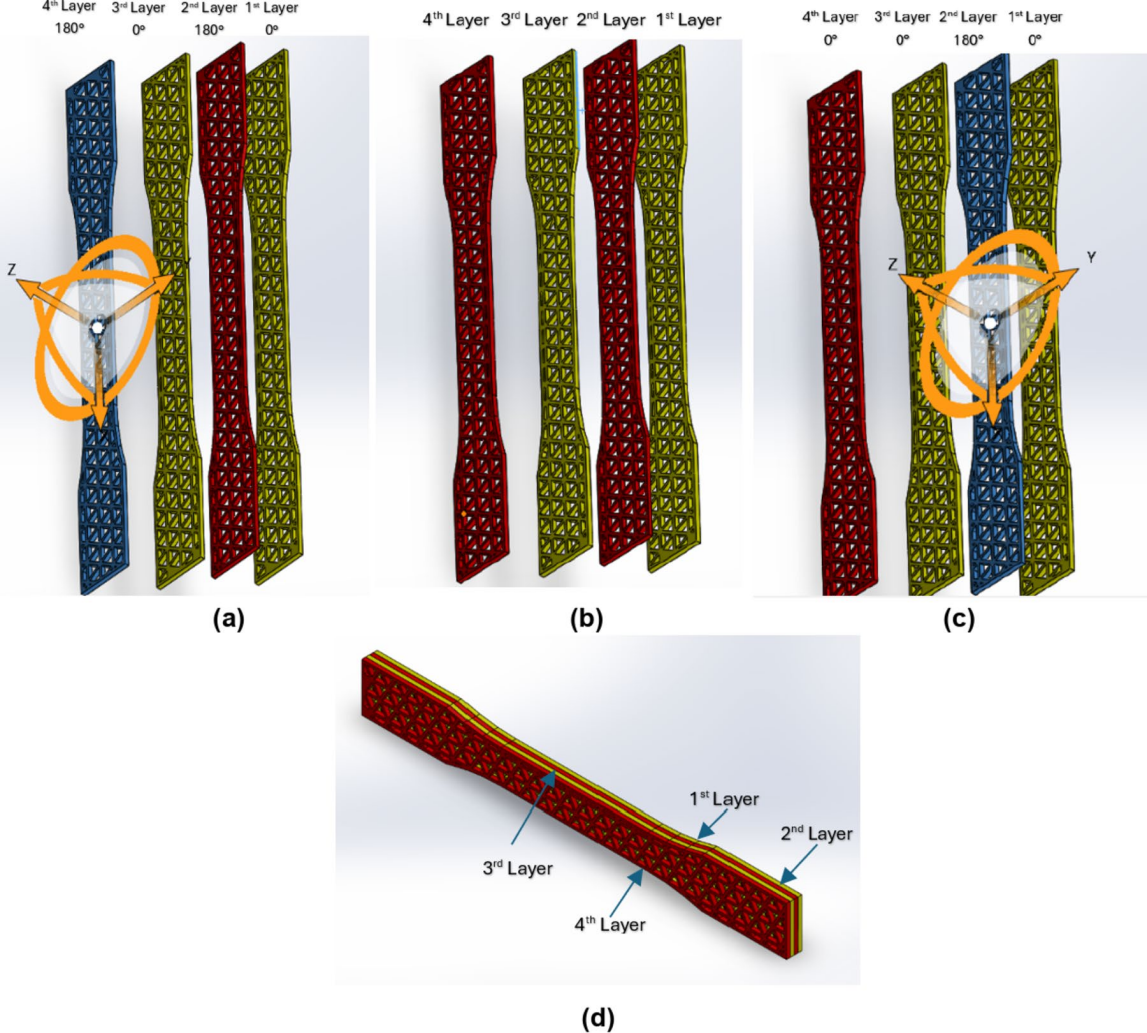
Similar pattern combination samples (4 Layers) (SolidWorks 2023) **(a)** Four Layers of the same pattern **(b)** Rotation of second layer 180 **(c)** Rotation of fourth layer 180° **(d)** Building of four layers (Final sample).

The third category is for the samples built with a combination of four layers of the same infill patterns. The first layer was built in one of the studied patterns; this layer is the reference one for the rest of the layers, which means it has a zero angle as a reference. The second layer has the same infill pattern but is built 180 degrees from the first layer and directly above it. The third layer has the same infill pattern but at zero degrees, which means it is in the same direction as the first layer and directly built above the second layer. Then finally, the fourth layer has the same infill pattern but is built 180 degrees from the first layer and directly above the third one, as shown in Fig. 3a, b and c, and 3d. After the layers are built, the four-layer combination specimens and their linking patterns will be the same as shown in Fig. 2d and e. After the specimens are designed, they are introduced to the 3D-printing machine software in the form of “STL files”.

**Table 1:** Process parameters used in printing

Printing Parameters	Value
Nozzle diameter	0.4 mm
Initial layer height	0.27 mm
Infill line width	0.4 mm
Top/Bottom thickness	0 mm
Printing temperature	210◦C
Build plate temperature	55◦C
Print speed	60 mm/s
Filament Flow	100%
Enable retraction	Yes
Travel speed	80 mm/s
Raster orientation	[0◦]
Printing orientation	Flat [y-z]
Enable cooling	yes

### Experimental set-up

Each experiment has both fixed and variable parameters. Fixed parameters mean there is no change during experiments. Table 1 presents the printing parameters fixed for all the specimens.

For each test, there are three factors; the number of layers (3 Levels: single layer, two-layer combination, and four-layer combination), the infill density (2 Levels: 20% & 50%), and the number of patterns (3 Levels: Honeycomb, triangular and grid).

The Taguchi method was used to design experiments which yielded eighteen experiments for each test (tensile and compressive tests).

The specimens are classified into three categories according to the number of layers. Each specimen was printed with a replication of three for each pattern, for a total of 108 specimens.

**Table 2:** Description of prepared.

Infill Density	Infill Pattern	Category	Description	Specimen Code
20%	Triangle	1st category	Single layer	S.T 20%
20%	Honeycomb	1st category	Single layer	S.H 20%
20%	Grid	1st category	Single layer	S.G 20%
50%	Triangle	1st category	Single layer	S.T 50%
50%	Honeycomb	1st category	Single layer	S.H 50%
50%	Grid	1st category	Single layer	S.G 50%
20%	Triangle	2nd category	Two layers	T.T 20%
20%	Honeycomb	2nd category	Two layers	T.H 20%
20%	Grid	2nd category	Two layers	T.G 20%
50%	Triangle	2nd category	Two layers	T.T 50%
50%	Honeycomb	2nd category	Two layers	T.H 50%
50%	Grid	2nd category	Two layers	T.G 50%
20%	Triangle	3rd category	Four layers	F.T 20%
20%	Honeycomb	3rd category	Four layers	F.H 20%
20%	Grid	3rd category	Four layers	F.G 20%
50%	Triangle	3rd category	Four layers	F.T 50%
50%	Honeycomb	3rd category	Four layers	F.H 50%
50%	Grid	3rd category	Four layers	F.G 50%

Three replicate specimens were tested for each condition to ensure statistically meaningful data. It allowed the calculation of standard deviations and the identification of any significant differences between groups, preliminary analysis indicated that three replicates were sufficient to observe consistent trends and draw meaningful conclusions regarding the influence of infill pattern, layer configuration, and infill density on the tensile and compressive strength of the 3D-printed specimens.

The specimens of the three categories for the three patterns (triangular, honeycomb, and grid) with different infill densities (20% and 50%) were printed to be subjected to tensile, and compressive tests to evaluate the consequence of these parameters on the mechanical properties of the three categories printed specimens. The layer-by-layer manufacturing process inherent to 3D-printing can induce anisotropic behavior in PLA, resulting in directionally dependent mechanical properties. To mitigate the potential influence of anisotropy, consistent loading direction relative to the printing orientation was maintained throughout the study. However, it is acknowledged that subtle variations in material properties due to anisotropy may still exist within the specimens. Variations in surface quality, such as surface roughness, delamination, and the presence of voids, can significantly influence the stress distribution and failure mechanisms during mechanical testing. To minimize the impact of these surface irregularities, consistent printing parameters, including extrusion temperature, printing speed, and infill density, were maintained throughout the study. Visual inspection was also conducted to identify and mitigate the presence of any significant surface defects. A summary of the specimen codes and their corresponding descriptions is provided in Table 2.

#### Tensile strength test

**Fig. 4:**
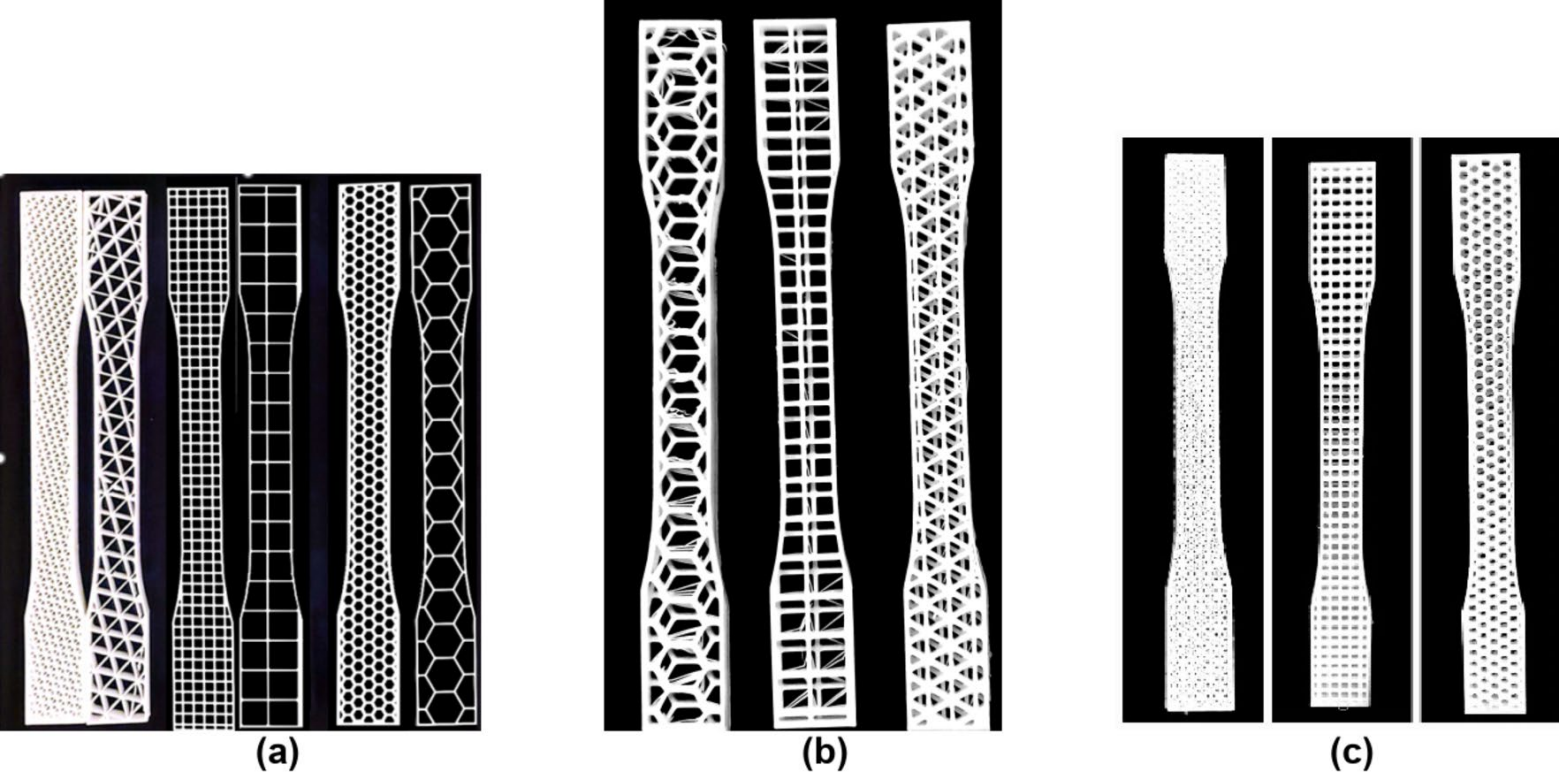
Printed Tensile Test Specimens **(a)** First-category specimens in 20% &50% infill **(b)** Second-category specimens in 20% infill **(c)** Second-category specimens in 50% infill.

Tensile strength tests were conducted on specimens conforming to ASTM D638 Type-I standards with a gauge length of 50 mm, gauge width of 13 mm, and a constant thickness of 5 mm. For single-layer specimens, the infill pattern occupied the entire 5 mm thickness. For two-layer specimens, each layer had a thickness of 2.5 mm, while for four-layer specimens, each layer was 1.25 mm thick. Figure 4 presents photographic images of the 3D-printed specimens, depicting both two- and three-layer configurations. These images were captured using a mobile phone and subsequently processed for enhanced clarity using the CamScanner application. Tensile tests were performed at room temperature (25.7 ± 1 °C) using a computerized Universal Testing Machine (capacity: 0.5 tons) at Ain Shams University, Cairo, Egypt. For tensile loading, the specimens were subjected to a uniaxial stress perpendicular to the printing direction. This orientation was selected to prioritize the evaluation of tensile strength within the individual layers while minimizing the potential influence of interlayer bonding weaknesses, which might be preferentially exploited under loading parallel to the printing direction. The specimen was positioned between the jaws of the testing machine with a grip distance of 115 mm, following ASTM D638. The test was performed at a crosshead speed of 2 mm/min. Strain percentage, ultimate tensile strength, and yield strength at the breakage are evaluated for each specimen at the end of the test.

#### Compression strength test

Compression tests were conducted on specimens prepared according to ASTM D695 standards, with dimensions of 12.7 mm length, 12.7 mm width, and 25.4 mm height. For single-layer specimens, the entire 25.4 mm height was occupied by the infill pattern. For two-layer specimens, each layer had a thickness of 12.7 mm, while for four-layer specimens, each layer was 6.35 mm thick. Compression tests were performed using a computerized Universal Testing Machine (capacity: 10 tons) at room temperature (25.7 ± 1 °C) at Ain Shams University, Cairo, Egypt. Specimens were subjected to uniaxial compressive loading until failure.

Perpendicular loading was employed to directly evaluate the compressive strength of the infill structures and the effectiveness of interlayer bonding in resisting compressive forces. Conversely, parallel loading could potentially induce significant shear stresses between layers, potentially leading to delamination and reduced compressive strength.

**Fig. 5:**
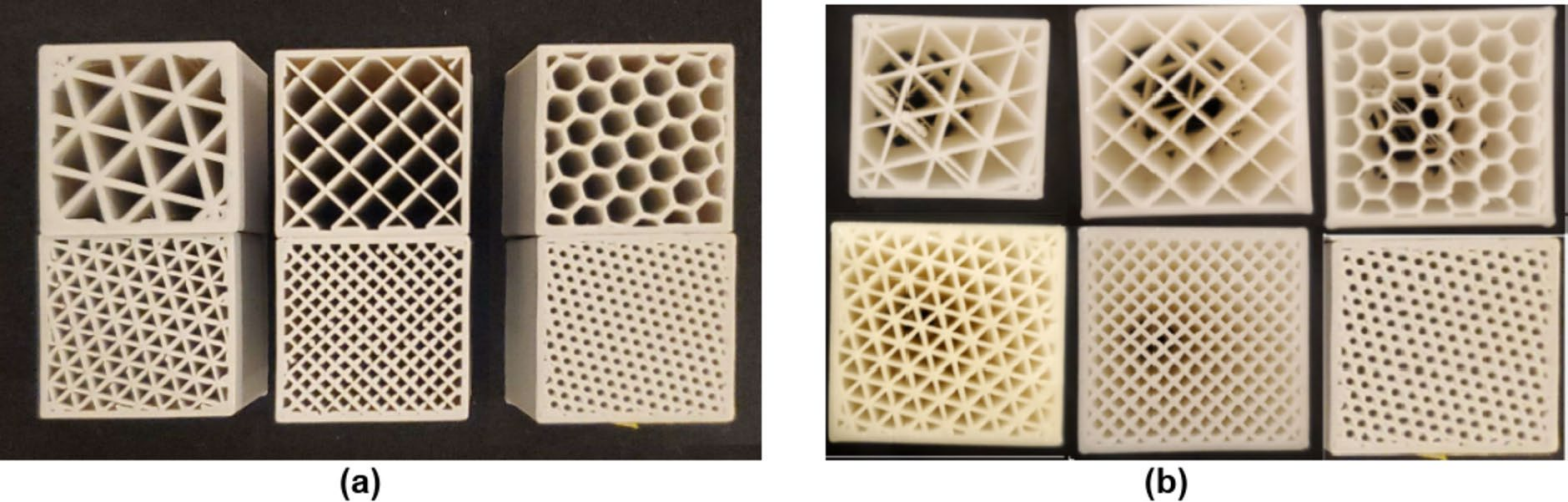
Printed Compression Test Specimen **a)** First category samples for 20% &50% b**)** Second & third category samples for 20%& 50% infill.

Figure 5 presents photographic images of the 3D-printed specimens utilized for the compression tests, depicting both the two-layer and three-layer configurations. These images were captured using a mobile phone and subsequently processed for enhanced clarity using the CamScanner application.

## Results and discussion

Fused filament fabrication processes can induce thermal and strain hardening within the extruded PLA filament, altering its molecular structure and potentially enhancing its mechanical properties^33^. The subsequent investigation focuses on the influence of key process parameters, including infill density (20% & 50%), infill patterns (triangular, honeycomb & grid), and the number of built layers, on the tensile and compressive strength of 3D-printed structures. A positive correlation between infill density and both tensile and compressive strength was observed across all three categories, with an increase in strength noted as infill density increased from 20 to 50%. This finding aligns with previous research in the field^19–22^.

Both tensile and compressive tests demonstrated that multi-layer configurations exhibited enhanced mechanical properties compared to their single-layer counterparts. This strength enhancement can be attributed to improved interlayer adhesion. The 180-degree rotation between adjacent layers likely increases the contact area between the infill structures, resulting in a larger bonding surface. This enhanced bonding facilitates more effective load transfer between layers, contributing to a more cohesive and robust structural integrity.

### Influence of process parameters on the tensile strength

**Fig. 6:**
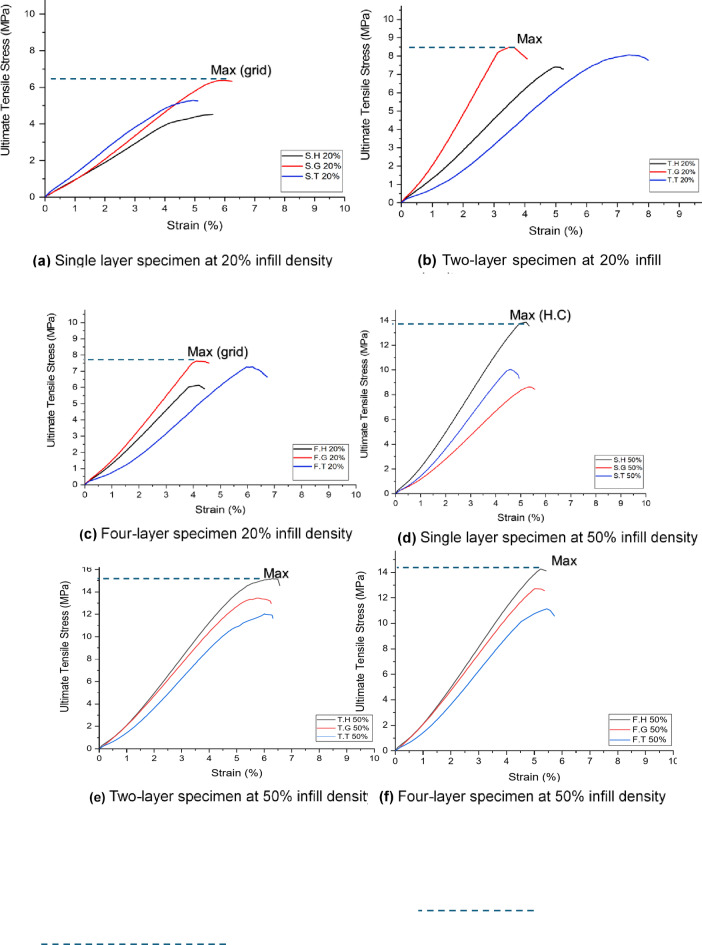
Tensile strength behavior of the three categories of specimens.

The tensile stress-strain behavior of 3D-printed specimens was investigated across varying infill densities (20% and 50%) and layer configurations (single, two, and four layers) for three distinct infill patterns: honeycomb, grid, and triangular. At 20% infill density, the grid pattern consistently exhibited the highest ultimate tensile strength, followed by triangular and honeycomb. Conversely, at 50% infill density, the honeycomb pattern demonstrated the highest strength, followed by triangular and grid. This trend was observed across all layer counts. Figure 6 illustrates the relationship between infill density and the number of built layers. Table 3 summarizes the tensile strength and strain variations observed between combined layers (two and four layers) compared to their single-layer counterparts.

**Table 3:** Tensile strength and strain percentage mutation between two- and four-layer specimens compared to single-layer specimens.

Single Specimen	Tensile strength (MPa)	Strain %	Combined Specimens	Tensile strength (MPa)	Strain percentage (%)	Tensile strength variation (%)	Variation in Strain (%)
S.T 20%	5.2895	5.0952	T.T 20%	8.0651	7.934	52%	56%
S.H 20%	4.5232	5.5999	T.H 20%	7.4088	5.2467	64%	-6%
S.G 20%	6.3829	6.2371	T.G 20%	8.3739	4.213	31%	-32%
S.T 50%	10.046	4.486	T.T 50%	12.042	6.0143	20%	34%
S.H 50%	13.862	5.0132	T.H 50%	15.157	6.1443	9%	23%
S.G 50%	8.6259	5.0172	T.G 50%	13.433	5.7318	56%	14%

**Single Specimen**	**Tensile strength (MPa)**	**Strain %**	**Combined Specimens**	**Tensile strength (MPa)**	**Strain percentage (%)**	**Tensile strength variation (%)**	**Variation in Strain (%)**
S.T 20%	5.2895	5.0952	F.T 20%	7.1844	6.7251	36%	32%
S.H 20%	4.5232	5.5999	F.H 20%	6.1419	4.4132	36%	-21%
S.G 20%	6.3829	6.2371	F.G 20%	7.6278	4.5789	20%	-27%
S.T 50%	10.046	4.486	F.T 50%	11.143	5.4525	11%	22%
S.H 50%	13.862	5.0132	F.H 50%	14.265	5.2289	3%	4%
S.G 50%	8.6259	5.0172	F.G 50%	12.72	5.3216	47%	6%

As evident from the presented data in Table 3, At 20% infill density, specimens with a grid pattern exhibited the highest ultimate tensile strength among the investigated geometries, suggesting superior load distribution and stress transfer within its structure. This outperformance compared to honeycomb and triangular patterns may be attributed to the inherent characteristics of the grid design. However, increasing the number of layers generally led to an improvement in tensile strength, particularly evident in the honeycomb and grid patterns. This trend can be explained by enhanced interlayer bonding and a more uniform stress distribution across the multiple layers. Notably, while the four-layer configuration offered an overall increase in tensile strength there is a slight drawback compared to two two-layer combinations, the introduction of numerous interfaces between layers may have introduced complexities. These complexities could potentially lead to localized stress concentrations or interfacial weaknesses, potentially influencing the overall mechanical performance.

At 50% infill density, the honeycomb pattern consistently demonstrated the highest ultimate tensile strength across all layer configurations. This observation can be attributed to the increased material volume fraction at this density, resulting in a significant increase in ultimate tensile strength for all patterns and layer counts. Interestingly, within the combined layer configurations (two and four layers), the grid pattern exhibited the most significant enhancement in tensile strength at 50% infill density compared to single layers. This superior performance aligns with the findings reported by Maqsood et al.^34^ and can be attributed to the grid pattern’s inherent structural characteristics. The interconnected nature of the grid facilitates efficient load transfer across multiple layers, forming a robust network that effectively distributes stresses throughout the structure.

The significant improvement (64%) in tensile strength observed for two-layer honeycomb structures at 20% infill density can be attributed to several factors. The open-cell geometry of the honeycomb pattern likely facilitates improved interlayer bonding at this lower infill density. The 180-degree rotation of the layers likely enhances this interlock, leading to a more cohesive structure. Additionally, the honeycomb structure’s inherent ability to distribute stresses effectively minimizes stress concentrations at the layer interfaces, further contributing to the observed strength enhancement. However, increasing the infill density can lead to a decrease in load transfer efficiency as the open cell structure becomes more filled, potentially hindering its performance that’s why at 50% infill density the best enhancement after combining layers was for the grid pattern (47%) not the honeycomb (3%) although the honeycomb was exhibit the maximum tensile strength.

**Fig. 7:**
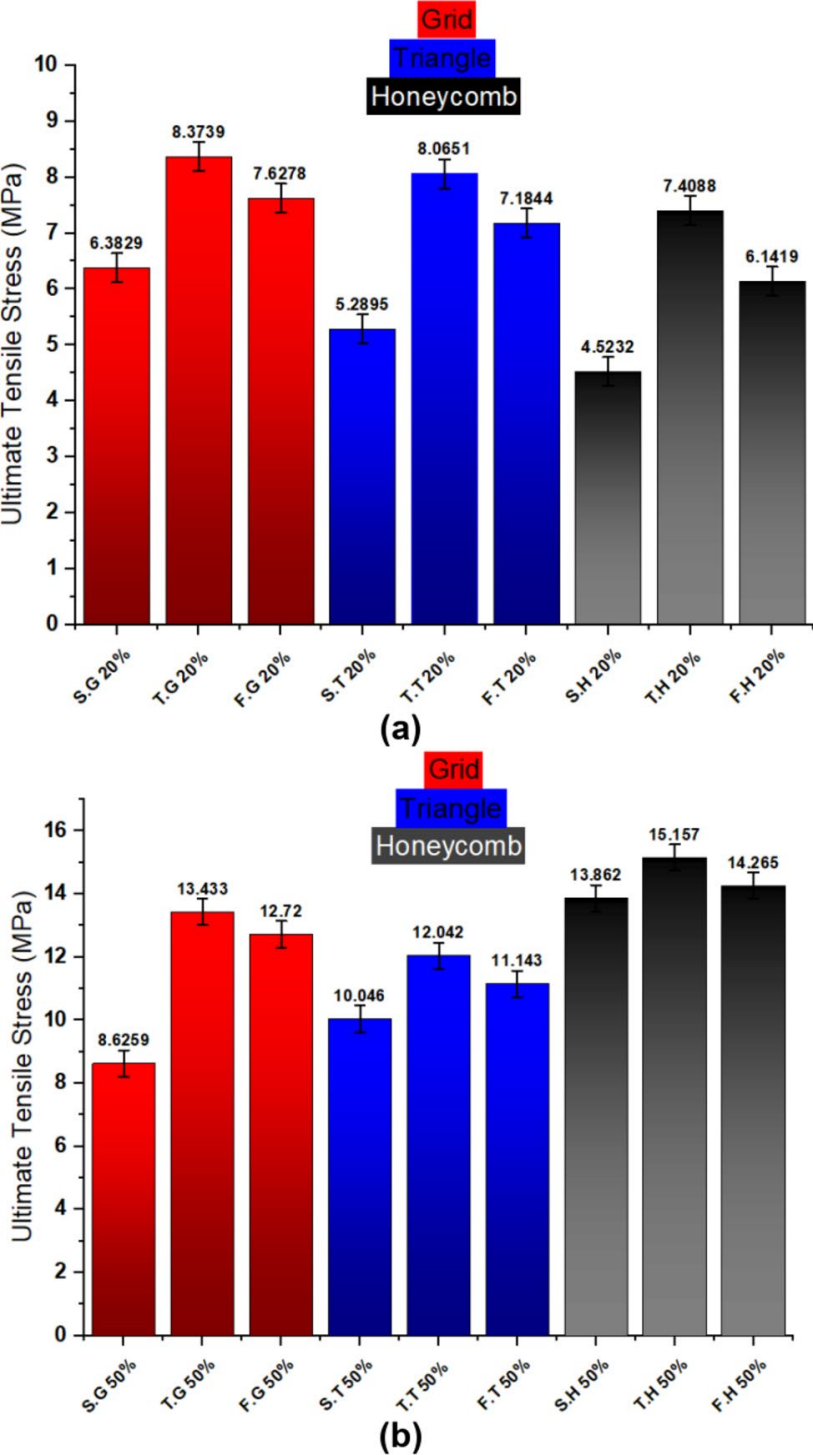
Comparison of tensile strength of three categories prepared specimens **(a)** 20% infill density **(b)** 50% infill density.

In a general way, The multi-layer configurations (two and four layers) effectively function as a form of internal reinforcement within the 3D-printed structures. The combination of layers, particularly with the 180-degree rotation employed, increases the effective cross-sectional area and enables more efficient load distribution through the interconnected infill structures. This enhanced load transfer mechanism leads to a significant increase in tensile strength compared to single-layer specimens, effectively mimicking the effect of higher infill densities. Notably, the two-layer and four-layer configurations at 20% infill density demonstrate mechanical behavior that is comparable to that of higher infill densities in single-layer specimens, highlighting the effectiveness of this multi-layer approach in enhancing material utilization and overall mechanical performance. Figure 7 shows a comparison of tensile strength for the three categories of prepared specimens where Fig. 7a shows the 20% infill density and Fig. 7b shows the 50% infill density.

### Influence of process parameters on compressive strength

**Fig. 8:**
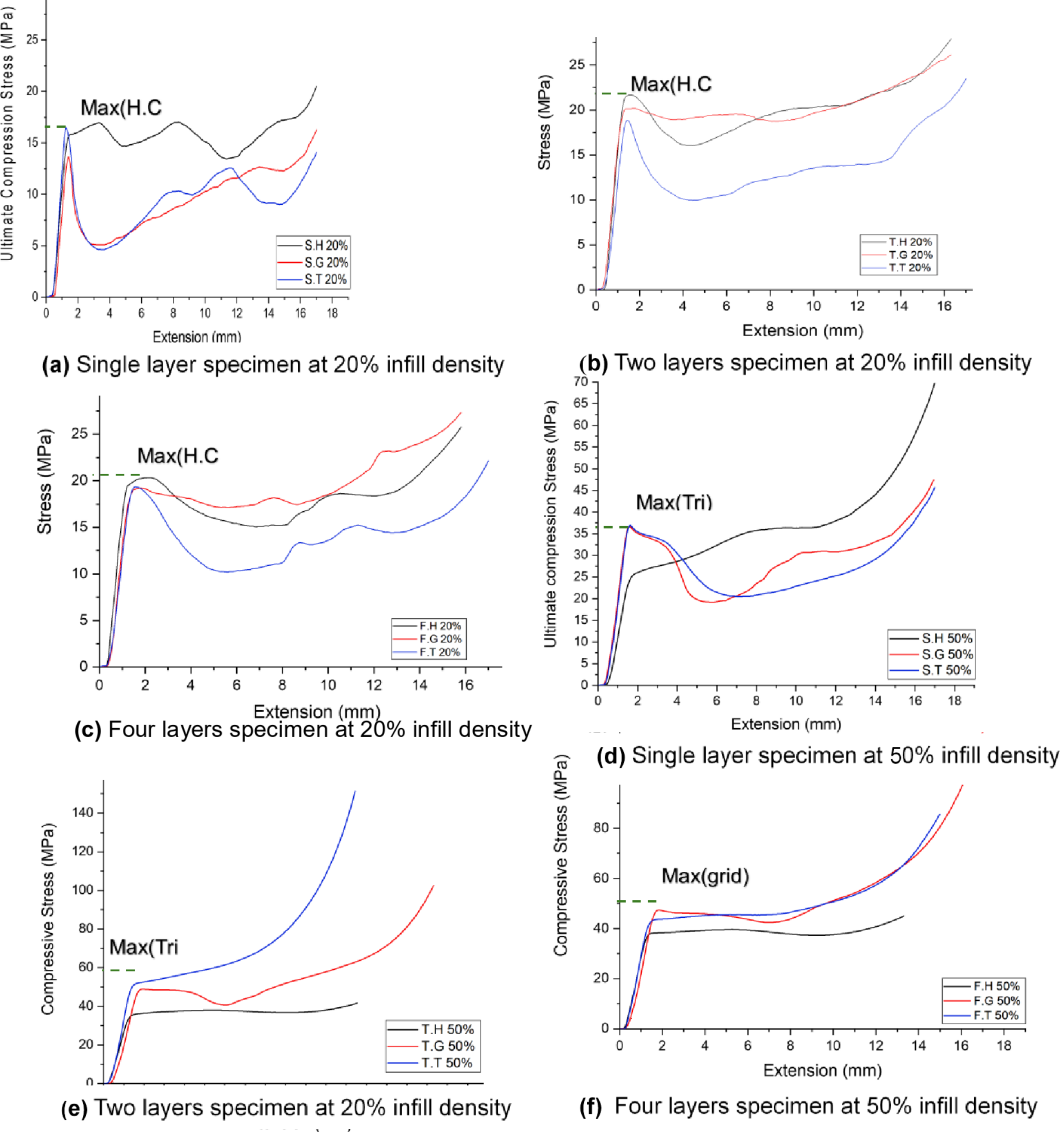
Compressive strength behavior of the three categories specimens.

Compression tests revealed significant variations in compressive strength across different infill patterns, layer configurations, and infill densities. Two-layer configurations consistently demonstrated higher compressive strengths compared to their single-layer counterparts across both 20% and 50% infill densities. While four-layer configurations demonstrated an increase in compressive strength, the magnitude of this enhancement was generally less pronounced than that observed for two-layer configurations. This may be attributed to factors such as increased interface complexity and potential localized stress concentrations, as previously discussed in the context of tensile behavior. All specimens exhibited ductile behavior during compression, characterized by significant plastic deformation prior to failure, a characteristic typical of PLA.as shown in Fig. 8. The results of the three categories of compressive strength and strain mutation between the combined layers (two and four-layer) compared to the single-layer specimens are listed in Table 4 briefly.

**Table 4:** Compressive strength mutation between two- and four-layer specimens compared to single-layer specimens.

Single layer Specimen	Compressive strength (MPa)	Extension (mm)	Combined Specimens (Two Layers)	Compressive strength (MPa)	Extension (mm)	Variation in compressive strength %	Variation in strain %
S.H 20%	16.972	3.341	T.H 20%	21.654	1.5756	28%	-6%
S.G 20%	13.687	1.4076	T.G 20%	20.166	1.747	47%	-32%
S.T 20%	16.516	1.2345	T.T 20%	19.358	1.4617	17%	56%
S.H 50%	36.393	9.4873	T.H 50%	38.033	5.4931	5%	23%
S.G 50%	36.639	1.5706	T.G 50%	48.502	2.9424	32%	14%
S.T 50%	36.935	1.6325	T.T 50%	52.007	1.7144	41%	34%

**Single Specimen**	**Compressive strength (MPa)**	**Extension** **(mm)**	**Combined Specimens(Four Layers)**	**Compressive strength** **(MPa)**	**Extension** **(mm)**	**Variation in compressive strength %**	**Variation in strain %**
S.H 20%	16.972	3.341	F.H 20%	20.336	2.088	20%	-21%
S.G 20%	13.687	1.4076	F.G 20%	19.224	1.8042	40%	-27%
S.T 20%	16.516	1.2345	F.T 20%	18.866	1.5761	14%	32%
S.H 50%	36.393	9.4873	F.H 50%	39.573	5.5556	9%	4%
S.G 50%	36.639	1.5706	F.G 50%	47.432	1.8602	29%	6%
S.T 50%	36.935	1.6325	F.T 50%	44.549	3.0417	21%	22%

Figures ** (a)** to **(f)** present the compressive stress-strain curves for 3D-printed specimens with varying infill densities (20% and 50%), layer configurations (single, two, and four layers), and infill patterns (honeycomb, grid, and triangular). All curves exhibit a characteristic non-linear response under compressive loading, with a distinct peak stress followed by a decline. Across all configurations of 20% infill density, the honeycomb infill pattern consistently demonstrated the highest peak compressive stress, indicating superior compressive strength compared to the grid and triangular patterns. This trend can be attributed to the inherent structural advantages of the honeycomb geometry, such as its interconnected cellular structure, which effectively distributes loads and resists compressive failure modes like buckling and shear band formation.

At 20% infill density, the honeycomb pattern consistently exhibited the highest compressive strength across all layer configurations. Moreover, a slight decrease in compressive strength was observed across all patterns with an increasing number of layers as a result of the reasons discussed before in tensile test results discussion.

At 20% infill density, the grid pattern exhibited the most significant enhancement in compressive strength at two-layer configuration (47%) and four-layer configuration (40%). This can be attributed to the inherent structural characteristics of the grid pattern. The interconnected nature of the grid likely provides a more stable and interlocking structure when combined with a second layer printed at 180 degrees. This interlocking effect may effectively distribute compressive loads across the layers, minimizing stress concentrations and enhancing load transfer between the layers. Followed by honeycomb then triangle patterns.

**Fig. 9:**
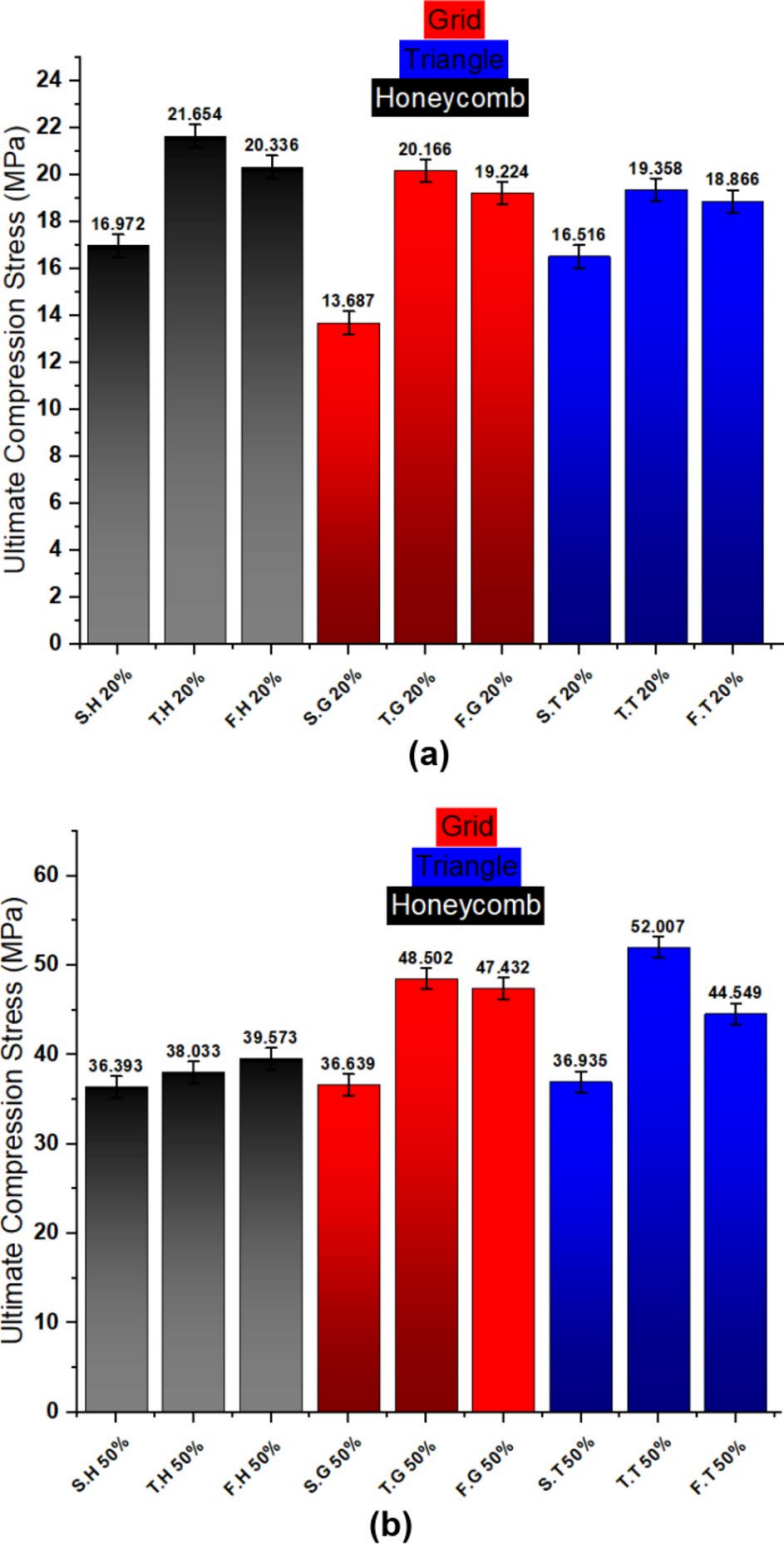
Comparison of compressive strength of three categories prepared specimens **(a)** 20% infill density **(b)** 50% infill density.

However, At 50% infill density, the triangular pattern exhibited the highest compressive strength in single-layer and two-layer configurations, likely due to its efficient load distribution and interlocking structure. However, in the four-layer configuration, the grid pattern demonstrated superior compressive strength. This may be attributed to the grid pattern’s enhanced interlayer bonding and more effective load transfer across multiple layers in the four-layer configuration. These findings suggest that the interplay between infill pattern, layer number, and infill density significantly influences the compressive strength of 3D-printed structures. Furthermore, the two-layer configuration resulted in the most significant compressive strength improvement for the triangular pattern (41%), followed by grid (32%) and honeycomb (5%), aligning with the findings of Maqsood et al.^35^. Conversely, the grid pattern exhibited the most pronounced enhancement (29%) in four-layer configurations, followed by triangular (21%) and honeycomb (9%). Table 4 provides quantitative data supporting these observations. Figure 9 shows a comparison of tensile strength for the three categories of prepared specimens where Fig. 9a shows the 20% infill density and Fig. 9b shows the 50% infill density.

## Taguchi analysis

**Fig. 10:**
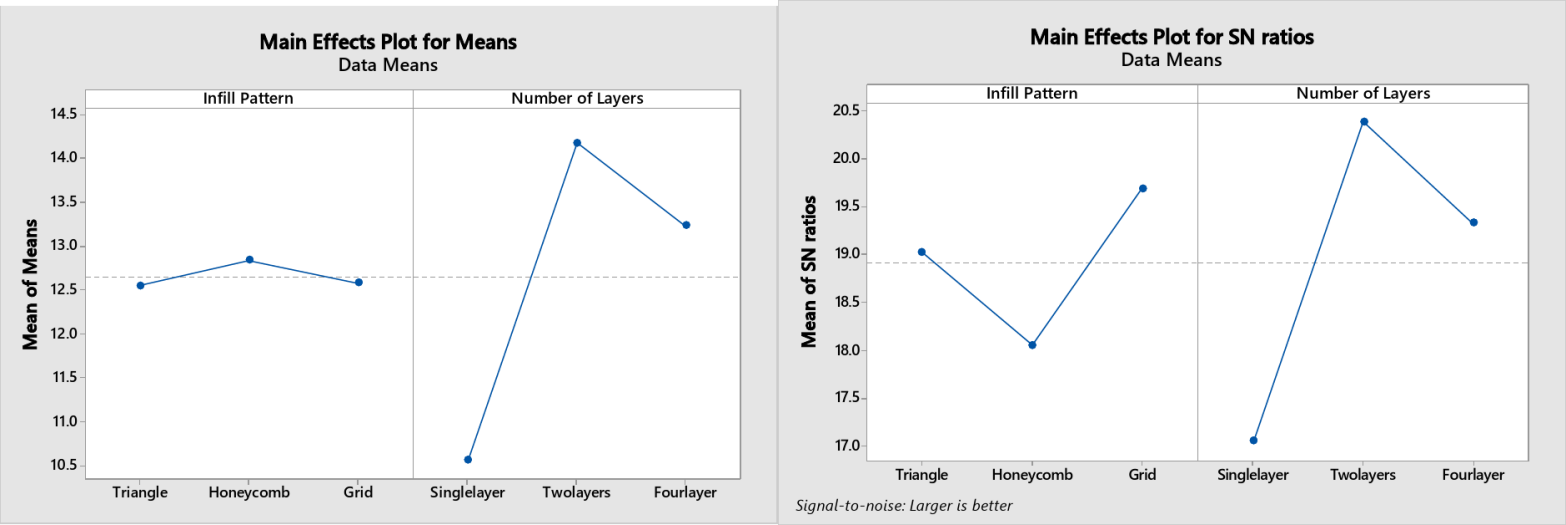
Taguchi optimization for the 20% infill density patterns.

**Fig. 11:**
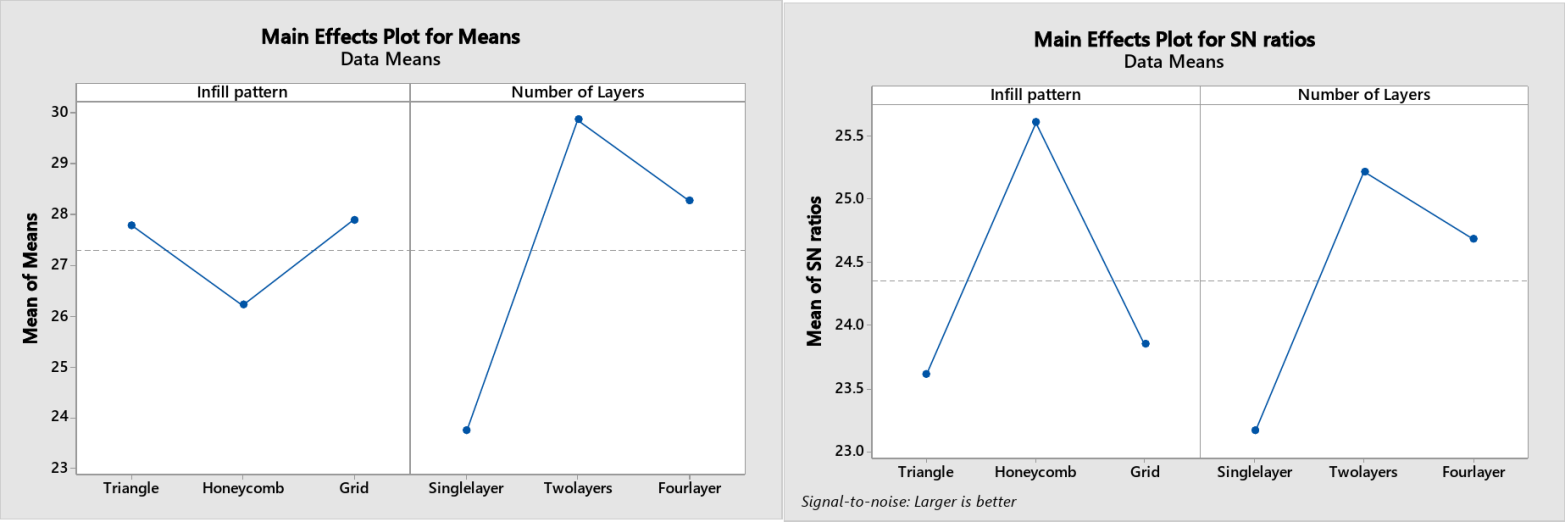
Taguchi optimization for the 50% infill density patterns.

The Taguchi method was employed to optimize the experimental design and identify the most influential factors affecting the tensile and compressive strength. An orthogonal array was utilized to systematically investigate the effects of [number of layers, infill density, and infill patterns] and their interactions while minimizing the number of experiments. The signal-to-noise ratio was calculated to determine the optimal combination of factor levels that yielded the most robust and consistent performance, guiding subsequent experimental decisions and process optimization efforts. Using Minitab Statistical Software, the analysis was performed for the two factors: the number of layers and infill pattern type at 20% infill density as shown in Fig. 10, and then at 50% density as shown in Fig. 11. The optimization criteria, “larger is better” is taken into consideration in the graphs of the plot for means, and signal-to-noise ratios (SN).

From the above figures, it is noticed that for both the 20% and the 50% infill patterns, the most significant factor in the mechanical properties was the infill print structure (singular, two & four layers) followed by the infill pattern. For the plot of means, the honeycomb pattern shows the highest values of the tensile and compressive strength with the two-layer combination structure for the 20% infill density, followed by the grid pattern and then the triangular pattern while the triangular pattern shows the highest values of the tensile and compressive strength with the two-layer combination structure for the 50% infill density, followed by the honeycomb pattern and then the grid pattern. The four-layer combination structure showed the second highest values of the mechanical properties as little bit as the two-layer combination, followed by the single-layer structure.

**Fig. 12:**
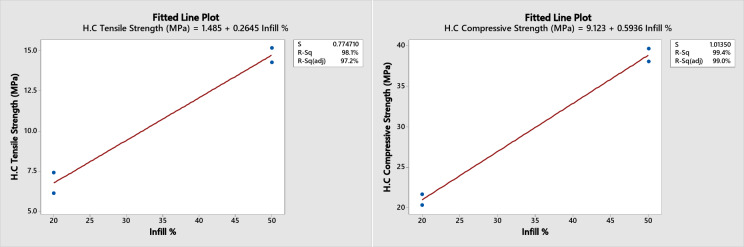
Honeycomb fitting plot for tensile and compression strength.

**Fig. 13:**
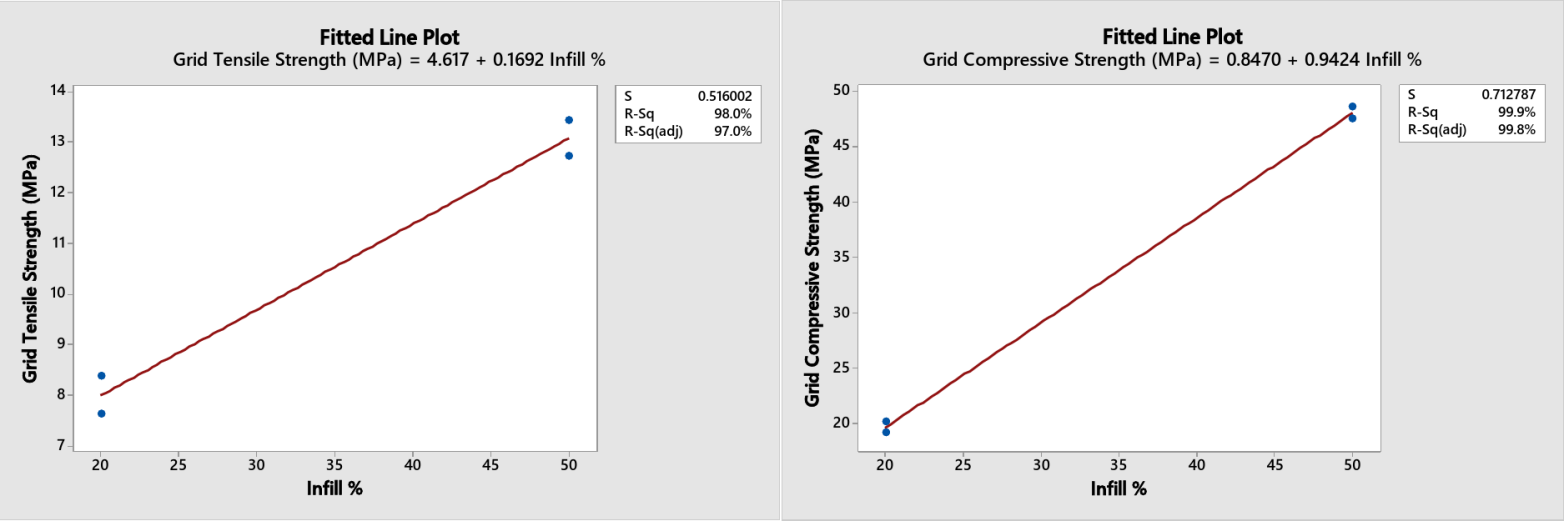
Grid fitting plot for tensile and compression strength.

**Fig. 14:**
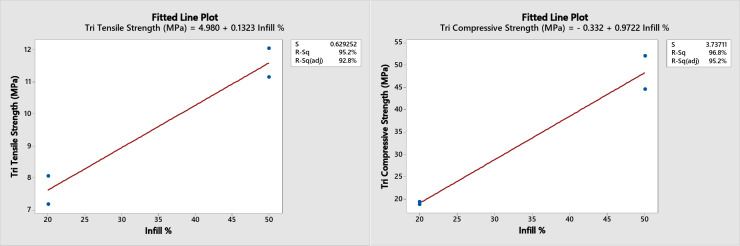
Triangular fitting plot for tensile and compression strength.

A regression analysis for the three patterns at the Minitab software was performed for the two and the four layers combination together as their results are too close to each other. The following predicted mechanical properties equations (shown in figures) were obtained with a range from 20 to 50% infill as shown in Figs. 12 and 13, and 14 with an error of approximately ± 2% for the tensile strength of the honeycomb, grid patterns and ± 5% for the triangular pattern.

## Conclusions

This study demonstrates that strategic manipulation of infill patterns and layer configurations significantly influences the mechanical properties of 3D-printed PLA structures. Multi-layer configurations, particularly with 180-degree layer rotation, exhibit enhanced tensile and compressive strength, offering the potential for high strength-to-weight ratios. Key findings include:

- **Tensile Strength**:
  - Grid patterns excel at 20% infill density in single-layer configurations.
  - Two-layer honeycomb configurations exhibit the most significant tensile strength improvement (64%).
  - Honeycomb patterns demonstrate the highest strength at 50% infill density.
  - Grid patterns show the most significant improvement at 50% infill density in two-layer configurations (56%).
- **Compressive Strength**:
  - Honeycomb patterns exhibit the highest strength at 20% infill density.
  - Grid patterns demonstrate the most significant improvement in two-layer configurations (47%).
  - Triangular patterns exhibit the highest strength and the most significant improvement at 50% infill density (41%).
- **Taguchi Analysis**:
  - The number of layers is the most significant factor influencing both tensile and compressive strength, followed by the infill pattern.
  - Honeycomb patterns are optimal at 20% infill density, while triangular and grid patterns perform best at 50% infill density.

This study offers avenues for weight reduction critical for applications such as aerospace and automotive components, leading to improved fuel efficiency and enhanced crashworthiness. Furthermore, this approach has implications for lightweighting in biomedical and consumer goods.

However, this study has limitations, including the lack of investigation into the influence of environmental factors such as temperature and humidity. Additionally, variations in interlayer adhesion due to inconsistencies in the 3D-printing process may have influenced the results.

Future research should investigate the influence of varying layer thicknesses, explore the performance of hybrid infill patterns, and evaluate the impact of different printing parameters and post-processing techniques such as heat treatment to further enhance the performance and optimize the 3D-printing process.

## Data Availability

the datasets generated and analyzed during the current study are available from the corresponding author upon reasonable request.
